# The Psychosocial Impacts of COVID-19 on a Sample of Australian Adults: Cross-sectional Survey and Sentiment Analysis

**DOI:** 10.2196/29213

**Published:** 2021-07-02

**Authors:** Jillian Ryan, Hamza Sellak, Emily Brindal

**Affiliations:** 1 Precision Health Future Science Platform Commonwealth Scientific and Industrial Research Organisation Adelaide Australia; 2 Nutrition and Health Program Health and Biosecurity Commonwealth Scientific and Industrial Research Organisation Adelaide Australia; 3 Data61 Commonwealth Scientific and Industrial Research Organisation Melbourne Australia

**Keywords:** COVID-19, natural language processing, behavioural science, NLP, behavior, impact, Australia, community, sentiment, data set, machine learning

## Abstract

**Background:**

The COVID-19 pandemic has had enormous impacts on people’s lives, including disruptions to their normal ways of behaving, working, and interacting with others. Understanding and documenting these experiences is important to inform the ongoing response to COVID-19 and disaster preparedness efforts.

**Objective:**

The aim of this study was to examine the psychosocial impacts of COVID-19 on a sample of Australian adults.

**Methods:**

The data analyzed were derived from a larger cross-sectional survey of Australian adults that was administered during the month of May 2020. Participants (N=3483) were asked in which ways COVID-19 had most greatly impacted them; the responses produced a text data set containing 1 COVID-19 impact story for each participant, totaling 86,642 words. Participants also completed assessments of their sociodemographic characteristics (sex, age, financial stress), level of concern related to COVID-19, personality trait profile, and satisfaction with life. Impact stories were analyzed using sentiment analysis and compared against the Theoretical Domains Framework to determine the most frequently impacted life domains. Finally, a multinomial regression analysis, stratified by participant sex, was conducted to identify the associations of psychological and demographic socializations with sentiment toward COVID-19.

**Results:**

In total, 3483 participants completed the survey, the majority of whom were female (n=2793, 80.2%). Participants’ impact stories were most commonly categorized as neutral (1544/3483, 44.3%), followed by negative (1136/3483, 32.6%) and positive (802/3483, 23.1%). The most frequently impacted life domains included behavioral regulation, environmental context and resources, social influences, and emotions, suggesting that the COVID-19 pandemic was impacting these areas of participants’ lives the most. Finally, the regression results suggested that for women, lower satisfaction with life and higher financial stress were associated with increased likelihood of negative, rather than positive, sentiment (*P*<.001); however, the proportion of variance in the sentiment that was explained was very small (<5%).

**Conclusions:**

Participant sentiment toward COVID-19 varied. High rates of neutral and negative sentiment were identified. Positive sentiment was identified but was not as common. Impacts to different areas of people’s lives were identified, with a major emphasis on behavioral regulation and related domains such as social influences, environmental context and resources, and emotions. Findings may inform the development of mental health and social support resources and interventions to help alleviate the psychosocial consequences of disaster response measures.

## Introduction

The COVID-19 pandemic is among the most disruptive and significant public health crises in recent human history. At the time of writing, nearly 3 million people had died from COVID-19 [1], while containment efforts had caused enormous upheaval to lives and livelihoods worldwide. In countries such as Australia, where viral spread has been relatively well controlled, the largest effects of the pandemic have been related not to the virus itself and its high mortality and infection rates, but to the containment measures needed to control outbreaks and how these measures have affected the community. People’s lives have changed dramatically, and adapting to the next phase of the pandemic and postpandemic life will require adjustment to a “new normal.” This means accepting a change of lifestyle in which alternative working arrangements, social distancing, and travel restrictions become the norm [2]. Adapting behavior can be challenging at the best of times, and in this instance, it is further complicated by feelings of loss related to letting go of previous ways of life [3]. The process of grief has been well documented, including nonlinear stages of disbelief, yearning, anger, depression, and acceptance [4]; however, more needs to be understood about the process and implications of the psychosocial impacts of COVID-19.

The virus that causes COVID-19, SARS-CoV-2, was first detected in Australia in January 2020. The Australian government’s approach to the mitigation of disease spread was strict and proactive, including significant restrictions on travel and public gatherings, quarantine protocols, and social distancing measures [5]. At the core of Australia’s public health response was a national shutdown that occurred during the months of March, April, and May 2020. This shutdown had the objectives of (1) delaying the impending epidemic to allow for resource planning to occur and (2) “flattening the curve” to reduce case numbers in Australia by minimizing the opportunity for disease transmission [5]. Shutdown measures consisted of a broad suite of restrictions, including the closure of gyms, pools, cinemas, and other health and entertainment facilities, a change to remote learning for universities and other higher education venues, restrictions on freedom to leave the house for nonessential reasons, restrictions on visitation to residential aged care facilities and hospitals, hygiene and social distancing measures, and the cancellation of events such as Australian and New Zealand Army Corp Day celebrations, arts, and sporting events [6].

The Australian government’s response to the pandemic was particularly strong compared to that of governments of other countries [7], and containment measures were enforced by law. In Western Australia, where the conditions are most stringent, individuals can face fines of up to Aus $50,000 (approximately US $38,000) or 12 months of imprisonment for breaching COVID mitigation rules. In other states, fines of between Aus $200 and $4000 (approximately US $152 and $3052) were issued [8]. Economic stimulus was provided to balance the effects of the containment measures. Stimulus efforts included the JobSeeker and JobKeeper programs, which effectively doubled income support payments and supplemented employees’ wages for businesses that were affected [9]. An additional Aus $1.1 billion (approximately US $841,313,000) was spent expanding mental health and telehealth services, increasing domestic violence services, and increasing food relief services [9].

Multicountry analyses suggest that Australia’s strict and proactive COVID-19 containment measures contributed to the delay and prevention of large infection outbreaks and to overall optimal outcomes [7]. However, they also imposed substantial limitations on Australians’ personal freedoms, livelihoods, and ability to maintain social connectivity. International travel effectively ceased due to the pandemic, with the number of citizens travelling outside of Australia plummeting to just 5050 individuals in May 2020, a >99% drop from the 798,700 departures recorded in May 2019 [10]. Similarly, domestic travel dropped from around 5 million trips in May 2019 to about 200,000 in May 2020 [11]. The shutdown also transformed ways of working, with only workers who were providing essential services (eg, emergency services, utilities), food and groceries, and health care permitted to travel to work. Australian research has documented significant declines in mental health, psychological distress, and contagion anxiety during the pandemic [12]; negative shifts in behavioral indicators of health, including physical activity, diet, sleep, alcohol consumption, and tobacco smoking [13]; and increases in food insecurity, with the strongest impacts felt by Australians with disabilities, those in rural areas, and others who face economic disadvantage or vulnerability [14,15]. COVID-19 is therefore a social crisis in addition to a public health crisis, and a greater understanding of the nature and extent of its impacts is needed [16].

Natural language processing offers a promising strategy to help understand the nature and impacts of complex health problems. Natural language processing uses intelligent computer algorithms to detect patterns and themes in unstructured data sets commonly containing text data [17]. A key advantage of this approach is the ability to automatically monitor or rapidly analyze unstructured data to identify and comprehend unanticipated or unforeseen health- and medical-related needs in the community (sometimes called infoveillance), monitor community sentiment toward health, and identify key geopolitical and psychosocial drivers for health-related behaviors [18]. Natural language processing can aid understanding of disease transmission and epidemic and pandemic trajectories by shedding light on community attitudes, behaviors, and experiences related to specific diseases. In particular, a substantial body of research has used sentiment analysis, which seeks to detect positive, negative, and neutral sentiments within text data, to understand aspects of the COVID-19 pandemic and in the context of other infectious diseases [19]. Broadly speaking, the aim of sentiment analysis is to classify words, sentences, or other units of text data according to the positive or negative polarity of that text [20]. These sentiment scores can then be interpreted in terms of the extent to which participant sentiment is favorable toward a focal issue, which can then inform more relevant and appealing messaging or identify intervention needs.

Natural language processing has a key advantage of being able to process large qualitative data sets rapidly and with greater objectivity than manual analysis. For example, it can help to identify community emotions toward different COVID-19 containment measures, identify geographical or sociodemographic correlates of vaccine hesitancy, and even detect outbreaks based on rapid analysis of social media data. In one example, researchers used machine learning to collect and analyze 86 million tweets published on the web-based social media platform Twitter in the United States to understand public sentiment toward COVID-19 and how it changed as the pandemic continued [21]. The results indicated an increasing volume of tweets over time and overall negative sentiment on the Twitter platform throughout the year.

Less explored is the potential application of natural language processing and sentiment analysis of research data that include populations representing a variety of community groups and that can be linked with demographic or other characteristics to better inform data-driven public health decision-making. Few studies have used this approach with purpose-collected research data sets and with research samples other than Twitter users [19]. Further efforts that use robust natural language processing techniques to identify and document both the expected (eg, shifts in health and travel behaviors, income streams) and unexpected impacts of containment measures are needed, as observational inferences are critical to optimizing public health response during the COVID-19 pandemic and in preparation for future public health and natural disasters. The purpose of this study is to use natural language processing to examine the breadth and nature of the impacts of the national shutdown on Australians during May 2020. More specifically, the study aimed to assess community sentiment toward the impacts of COVID-19, explore the nature of the impacts of COVID-19 and which life domains are most commonly affected, and identify psychological (personality traits, COVID-19–related concerns, and satisfaction with life) and sociodemographic characteristics (sex, age, and financial stress) that predict sentiment toward the impacts of the COVID-19 pandemic.

## Methods

The data analyzed in this study were collected as part of a larger cross-sectional survey of Australian adults that was conducted in the month of May 2020. Full details of the study methodology and primary findings have been published elsewhere [22]. The study received ethics approval from the Commonwealth Scientific and Industrial Research Organization Human Research Ethics Low-Risk Committee (LR2020/026), and all participants provided informed consent prior to completing the survey.

### Procedure

Convenience sampling methods were used in which a web-based survey was distributed to an email list of participants in a health and well-being program who had consented to being contacted about future studies or other tools relating to health and diet. In general, the list of contacts contained a higher proportion of women, and the members were slightly older and more educated in comparison with the general Australian population. Each member on this list was sent an email that included an invitation to participate and a link to the web-based survey and informed consent process.

### Materials

The web-based survey assessed the participants’ sociodemographic characteristics (sex and age), COVID-19 impacts, personality traits, and subjective well-being. COVID-19 impact stories, which formed the basis for the machine learning analysis, were collected via a single open-ended survey item. This item asked participants to finish the following sentence: “The COVID-19 outbreak has most greatly impacted…”

Participants’ psychosocial and demographic characteristics were also captured via self-report survey items. COVID-19–related financial stress was captured by a single item that asked participants to consider any financial stress they might have experienced during the COVID-19 pandemic and rate the extent to which they were unsure how they would pay upcoming bills on time. Responses were recorded on a 7-point Likert-type scale and ranged from 1, strongly disagree, to 7, strongly agree. The 14-item COVID-19 Concerns Scale was included to assess the participants’ concerns related to COVID-19. This scale asks respondents to indicate the extent to which they are concerned about different aspects of the pandemic, such as becoming infected with COVID-19, losing a job, or isolation from friends and family members [23]. Responses are recorded on a 4-point scale ranging from 1, to a great extent, to 4, not at all, with “I don’t know” also included as an option. The participants’ personality traits were captured by the validated Big Five Inventory-2-S [24], which assesses the “big five” personality traits: openness to experience, conscientiousness, extraversion, agreeableness, and neuroticism. This scale consists of 30 items, with responses captured on a 5-point Likert scale ranging from 1, strongly disagree, to 5, strongly agree. Finally, life satisfaction was assessed by the Satisfaction With Life Scale [25], which consists of 5 items that are measured on a 7-point Likert scale ranging from 1, strongly disagree, to 7, strongly agree. The scale is a validated measure that captures global life satisfaction and has adequate psychometric properties [25].

### Data Analysis

The survey was attempted by 4313 individuals; of these, 3483 answered the COVID-19 impact open-ended question (80.3% completion rate) required for the current study. Qualitative data were analyzed to detect sentiment and key themes using advanced natural language processing tools. We used the Stanford CoreNLP sentiment annotator [26], a machine learning model that uses recursive neural networks to perform sentiment analysis and classify input text on a 5-point scale of very negative, negative, neutral, positive, and very positive (higher scores indicate more positive sentiment). In addition, we analyzed data using the Valence Aware Dictionary and Sentiment Reasoner (VADER), a lexicon and rule-based sentiment analysis tool that produces a unidimensional measure of sentiment for a given sentence that reflects a summed score for each word in that text [27]. VADER and Stanford CoreNLP rely on distinct mechanisms that enable each tool to provide a different perspective on the data, with a core difference being that Stanford CoreNLP uses discrete but more detailed sentiment categories (5 sentiment levels) and VADER uses continuous numerical sentiment score values (continuous values that are more suitable to plotting) [28]. VADER produces summed sentiment scores that are normalized between –1 (most extreme negative) and +1 (most extreme positive). We used VADER sentiment scores to produce a plot depicting the distribution of sentiment and Stanford CoreNLP scores in subsequent analysis as a way to categorize the participants’ impact scores from very positive to very negative. The sentiment analysis results were manually inspected, and overall, the classifications appeared to be consistent with our expectations (see Multimedia Appendix 1).

Following text preprocessing, we automatically extracted the most frequent terms to identify themes in the data set, consistent with previous research [20]. The most frequently occurring words impacted the themes used in further analysis, as content themes and were manually classified according to the Theoretical Domains Framework [29]. This model acknowledges 14 life domains that are relevant to understanding and changing human behavior: knowledge, skills, social/professional role and identity, beliefs about capabilities, optimism, beliefs about consequences, reinforcement, intentions, goals, memory, attention and decision processes, environmental context and resources, social influences, emotions, and behavioral regulation. The Theoretical Domains Framework comprises established standardized terminology and organizing constructs for use in exploration, prediction, and intervention of human behavior. This framework is of particular value as a comprehensive framework that allows for the contextualization of different personal, interpersonal, and environmental influences on behavior and mood. COVID-19 impacts are therefore presented as frequencies with quotes provided as examples.

To test associations between participant characteristics and sentiment scores, an ordinal regression procedure was commenced. However, during the process, it became evident that our data did not meet the assumption of proportional odds. Therefore, multinomial logistic regression was undertaken to examine whether age, financial stress, life satisfaction, and personality traits (openness to experience, conscientiousness, extraversion, agreeableness, and neuroticism) were predictive of sentiment score. Analyses were stratified by gender. Because the very positive and very negative cells comprised fewer than 25 cases each, we merged them with the positive and negative cells, respectively, to arrive at 3 categories of the dependent variable: positive, neutral, and negative sentiment. The data were first examined to check that they met the model assumptions of an absence of multicollinearity based on variance inflation factors and tolerance, linearity to the logit, and absence of outliers [30,31]. These assumptions were met, and the multinomial regression could be conducted.

Variables were selected for inclusion in the model based on theoretical background knowledge [32]. There is a large body of research that demonstrates significant associations between individual differences in factors such as sociodemographic characteristics, personality trait profiles, and subjective well-being with psychosocial well-being outcomes [33]. Furthermore, insights into these associations have potential health communication applications, as they can inform the development of tailored public health interventions or identify in-need target audience segments within larger populations [34]. As this was an exploratory analysis with a primary aim to deduce novel insights from the data rather than to test a prespecified hypothesis, we included all variables of interest in the final regression model to determine each variable’s strength of association relative to the other variables included in the model. Continuous and ordinal variables of age, openness, conscientiousness, extraversion, agreeableness, neuroticism, satisfaction with life, and financial stress were entered as covariates [30]. Positive sentiment scores were set as the reference category such that the results reflect the likelihood of obtaining a neutral or negative sentiment score in comparison with a positive score. Statistical significance was deemed to be reached when α<.05, and standardized beta weights with 95% confidence intervals have been reported.

## Results

The majority of the participants were female (see Table 1). The participants’ mean age was 57.1 years (SD 12.2), and participants reported an average COVID-19–related financial stress score of 1.9 (SD 1.5) out of 7, with lower scores equating to less financial distress.

The qualitative data set contained 3483 COVID-19 impact stories, comprising a total of 86,642 words. The average length of the impact stories was 25 words; however, men provided shorter stories on average (mean 18.4, SD 24.8) compared to women (mean 26.4, SD 27.3).

**Table 1:** Participant characteristics among a sample of Australian adults in a cross-sectional study investigating the psychosocial impacts of COVID-19 in May 2020 (N=3483). For all variables, a higher score indicates a higher level of that variable.

Characteristic	Value, mean (SD)		
	Male participants (n=417)	Female participants (n=2793)	Overall sample (N=3483)
Age	61.8 (12.4)	56.2 (12.0)	57.1 (12.2)
Sentiment^a^	2.9 (0.7)	2.9 (0.8)	2.9 (0.8)
Financial stress^b^	1.9 (1.5)	1.9 (1.5)	1.9 (1.5)
Satisfaction with life^b^	4.4 (1.4)	4.1 (1.5)	4.2 (1.5)
COVID-19–related concerns^c^	2.5 (0.5)	2.6 (0.5)	2.6 (0.5)
Openness to experience^a^	3.5 (0.6)	3.6 (0.7)	3.6 (0.7)
Conscientiousness^a^	3.7 (0.7)	3.8 (0.7)	3.8 (0.7)
Extraversion^a^	3.1 (0.7)	3.0 (0.7)	3.1 (0.7)
Agreeableness^a^	3.7 (0.6)	4.0 (0.6)	4.0 (0.6)
Neuroticism^a^	2.5 (0.8)	2.7 (0.9)	2.7 (0.9)

^a^Scores range from 1 to 5.

^b^Scores range from 1 to 7.

^c^Scores range from 1 to 4.

### Sentiment Toward COVID-19 Impacts

**Figure 1:**
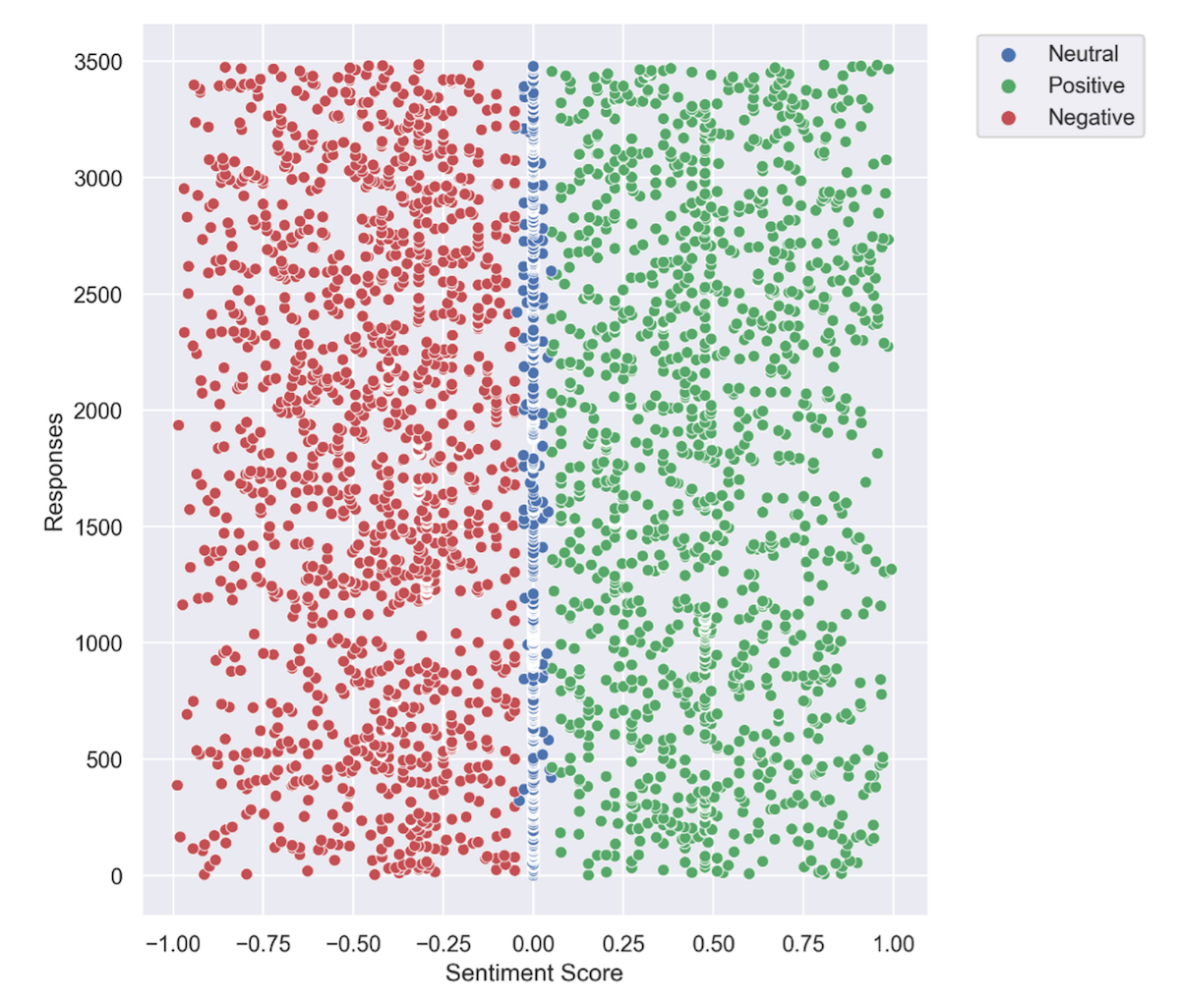
Distribution of standardized Valence Aware Dictionary and Sentiment Reasoner (VADER) sentiment scores in COVID-19 impact stories reported by a sample of Australian adults during May 2020.

The average Stanford CoreNLP sentiment score fell into the neutral category (mean 2.91, SD 0.76). Neutral classifications were most common, representing 44.3% of impact stories (n=1544/3483). There was also a relatively balanced prevalence of negative (n=1136/3483, 32.6%) and positive (n=802/3483, 23.1%) sentiment scores. Approximately 1% (n=38/3483) of all COVID-19 impact stories were categorized at the extreme ends of the sentiment distribution (very negative or very positive). The distribution of the VADER sentiment scores is depicted in Figure 1. Representative quotes from the data that exemplify each of the sentiment score categories are provided in Multimedia Appendix 1.

#### Nature of COVID-19 Impacts Across Different Life Domains Based on Word Frequency Analysis

The numbers and proportions of most frequently appearing words and their respective life domains are displayed in Table 2. The impacts were most commonly located within the four life domains of behavioral regulation, environmental context and resources, emotion, and social influences.

**Table 2:** Categorization of most frequently appearing words by theoretical life domain among impact stories from Australian adults in a cross-sectional study investigating the psychosocial impacts of COVID-19 in May 2020 (N=3483). Note: the theoretical life domains of skills, optimism, reinforcement, intentions, goals, and memory, attention, and decision processes were not identified in this data set.

Theoretical life domain	Top 50 words categorized into each domain, n (%)
Behavioral regulation	16 (32)
Environmental context and resources	13 (26)
Social influences	6 (12)
Emotion	6 (12)
Social/professional role and identity	4 (8)
Beliefs about consequences	3 (6)
Knowledge	1 (2)
Beliefs about capabilities	1 (2)

#### Behavioral Regulation

Impacts occurred primarily in the life domain of behavioral regulation, which considers ability and changes in ability to perform different behaviors (see Table 2), including health behaviors (eg, physical activity, eating vegetables), social behaviors (eg, spending time with friends and family), and risk behaviors (eg, drinking alcohol). Key themes within this category related to how the COVID-19 pandemic influenced participants’ abilities to fulfil their normal routines, with a focus on health and social behaviors, visiting others and travel, and use-of-time impacts. The closure of gyms and other health facilities was a particularly prevalent theme impacting participants’ ability to maintain physical and social health behaviors. This impact was compounded among respondents who reported special needs for supervised exercise, pools, or other equipment that was not easily substitutable with virtual or home-based exercise, including older adults and people with disabilities, injuries, and postoperative rehabilitation needs. More generally, the health and fitness impacts of gym closures were closely intertwined with loss of social opportunities and isolation. Restrictions on travel was also a key theme within this life domain; it was related to the ability to travel for work and to visit and support friends and family. Positive impacts related to increased flexibility for employees, such as ability to work from home and reduced time commuting. Example quotes from the data are provided below.

We have been unable to continue activities that are important to our health and fitness because of the closure of heated pools, massage clinics and other facilities for exercise suitable for aged persons with joint disabilities.

Female, 72 years old, 3/5 (neutral) sentiment score

It's massively affected my relationship with food. The relationship was already bad, but social isolation and gyms closing were just the LAST thing I needed. it couldn't have come at a worse time.

Female, 21 years old, 2/5 (negative) sentiment score

The COVID-19 outbreak has meant I am now working from home, which has been incredible for my mental health and wellbeing. I normally commute 2 hours per day, 3 times a week, and this was really taxing on me physically and emotionally. Working from home is so much more peaceful and stress-free. I am also the kind of person who benefits from working solo, so not being in the office environment has also been really positive for me. I was unable to get this opportunity before COVID-19, and only had 2 days a week approved to work closer to home. I now feel more confident to ask my employer for more days working from home.

Female, 34 years old, 4/5 (positive) sentiment score

#### Environmental Context and Resources

The environmental context and resources life domain considers external factors that facilitate or inhibit the development and expression of abilities, independence, social competence, and adaptive behavior. Common impacts falling under this theme are related to changes in the home environment, lost opportunities to have contact with or care for loved ones and friends, and difficulties acquiring groceries. The shutdown restrictions served as a major source of psychological distress and boredom for participants, particularly in relation to a lack of variation in environment, with work, socializing, and education of children now occurring primarily within the home location. Environmental constraints were also linked to behavioral outcomes (eg, changes in ability to eat healthily and exercise) as well as emotional outcomes such as stress, boredom, and anxiety. Some positive impacts were also noted as a result of fewer social obligations and reduced face-to-face contact.

Mental health has been an issue as I feel we at home are just getting through the weekly routine of trying to get some work done while helping our son to home school and then planning the next grocery shop. No real fun as we live in a dull routine.

Male, 43 years old, 2/5 (negative) sentiment score

My husband has dementia and is in a Nursing Home which is in total lockdown and I have not been able to have any contact with him since March 20th. I am devastated by this. Prior to lockdown I visited him daily. He continues to ask for me each morning and has no understanding as to why I no longer visit him.

Female, 77 years old, 2/5 (negative) sentiment score

I enjoy people social distancing from me when I am out and like that shops are clean. I prefer catching up with people online rather than in person.

Female, 41 years old, 4/5 (positive) sentiment score

#### Emotion

The third most commonly impacted life domain was emotion, which relates to individuals’ cognitive and psychological reactions and ways of coping with events and circumstances. Salient themes within this domain included stress, mental health, and anxiety, with the descriptors of “loss” and “hard” featuring prominently. Positive impacts included increased feelings of neighborhood cohesion and reductions in stress related to shorter commutes and greater hygiene and social distancing. Positive impacts tended to relate to reduced life “busyness” and a more relaxed pace.

My aunty past [sic] away. She was 84 and in a nursing home. Even though we (and the nursing home) knew she would pass away soon we were not allowed to visit in the last 2 weeks as the nursing home was in lock down. The funeral was limited to 10 people and we had to social distance. It was not the way we wanted to celebrate her life. I have had a 20% pay cut which is nothing compared to the sadness I feel with regard to not being able to see my aunty and say goodbye.

Female, 47 years old, 2/5 (negative) sentiment score

It has greatly reduced the traffic in my street. So many more neighbours are out and about, riding their bikes, walking with family, working in their gardens, with time to stop and chat (properly socially distanced of course). We all make way for others on the footpaths, and say hello or wave as we pass. I feel we have a more cohesive community spirit in our neighbourhood.

Female, 68 years old, 4/5 (positive) sentiment score

Cancelled holidays. Limited socialisation with friends. Worry about maintaining health and well-being (i.e. not getting infected with Covid-19).

Male, 60 years old, 2/5 (negative) sentiment score

Life has been less hectic and more relaxed. I have enjoyed not having any outside of my house responsibilities.

Female, 61 years old, 3/5 (neutral) sentiment score

#### Social Influences

The fourth life domain that was commonly impacted for the participants was social influences, referring to how people’s relationships with others affect their own thoughts, feelings, and behaviors. Themes within this category primarily related to friends and family members, particularly children.

Life is unrecognisable. Husband stood down from Qantas, children bored and under stimulated by home schooling, children's sport was a big part of all our lives. Depressed.

Female, 47 years old, sentiment score 2/5 (negative)

The pandemic had had more effect on my children and husband than me. My five year old has started experiencing anxiety in the night and sometimes cannot sleep. My other child has also become much more emotional.

Female, 42 years old, 2/5 (negative) sentiment score

With children aged 4 & 2, I have enjoyed being home with them. No pressure for play dates, outings etc.

Female, 37 years old, 4/5 (positive) sentiment score

### Psychosocial and Demographic Characteristics That Predict Sentiment Toward COVID-19

Associations between participant characteristics and COVID-19 sentiment were analyzed using regression models (stratified by sex). Model 1, which tested the association between female participants’ characteristics and sentiment scores, was statistically significant (χ^2^_18_=75.8, *P*<.001) (see Table 3). The model explained 3.2% (Nagelkerke *R*^2^) of the variance in sentiment (very small effect size). Of the variables included in the model, satisfaction with life, openness to experience, and financial stress were statistically significant predictors of sentiment. More specifically, for each 1-unit increase in satisfaction with life, there was a decrease in the odds of having a negative sentiment score rather than a positive sentiment score. Furthermore, for each 1-unit increase in openness to experience, the risk of reporting a negative relative to a positive sentiment score decreased by a factor of 0.832. Finally, for each 1-unit increase in financial stress, the odds of having a neutral rather than positive sentiment score increased by 1.128, and the odds of have a negative rather than positive sentiment score increased by 1.194.

Model 2, evaluating the association between participant characteristics and sentiment scores for men, was not statistically significant (χ^2^_18_=19.5, *P*=.36).

**Table 3:** Multinomial logistic regression identifying significant associations with COVID-19 sentiment among a sample of Australian adults reported in May 2020.

Characteristic		Positive sentiment, mean (SD)	Neutral sentiment					Negative sentiment					
			Mean (SD)	β	95% CI (lower)	95% CI (upper)	Mean (SD)		β	95% CI (lower)	95% CI (upper)		
**Model 1 (female participants)**													
	Age	56.8 (11.7)	56.1 (12.2)	0.998	0.990	1.007	56.0 (11.9)		0.998	0.989	1.007		
	Financial stress	1.65 (1.2)	1.9 (1.5)	*1.128* ^a^	1.039	1.224	2.1 (1.6)		*1.194*	1.100	1.297		
	Satisfaction with life	3.2 (1.1)	3.0 (1.0)	0.923	0.832	1.024	2.9 (1.1)		*0.832*	0.747	0.927		
	COVID-19–related concerns	2.6 (0.5)	2.6 (0.5)	0.931	0.753	1.152	2.6 (0.5)		1.019	0.815	1.274		
	Openness to experience	3.6 (0.7)	3.5 (0.7)	*0.823*	0.710	0.953	3.7 (0.7)		1.039	0.889	1.213		
	Conscientiousness	3.9 (0.7)	3.8 (0.7)	0.977	0.838	1.139	3.8 (0.7)		0.939	0.800	1.103		
	Extraversion	3.1 (0.7)	3.0 (0.7)	0.915	0.791	1.059	3.0 (0.8)		0.902	0.774	1.051		
	Agreeableness	4.0 (0.6)	4.0 (0.6)	0.981	0.824	1.168	4.0 (0.6)		1.101	0.915	1.324		
	Neuroticism	2.6 (0.9)	2.7 (0.9)	0.982	0.856	1.127	2.7 (0.9)		0.991	0.858	1.145		
**Model 2 (male participants)**													
	Age	61.8 (14.5)	63.1 (11.0)	1.007	0.983	1.032	59.6 (13.1)		0.985	0.960	1.011		
	Financial stress	1.8 (1.4)	1.8 (1.5)	1.015	0.821	1.254	2.1 (1.6)		1.044	0.832	1.311		
	Satisfaction with life	3.2 (1.0)	3.2 (1.0)	0.933	0.677	1.287	3.0 (1.1)		0.760	0.533	1.084		
	COVID-19–related concerns	2.6 (0.5)	2.5 (0.5)	0.680	0.388	1.189	2.6 (0.5)		0.975	0.518	1.833		
	Openness to experience	3.4 (0.6)	3.5 (0.6)	1.244	0.781	1.983	3.4 (0.6)		1.141	0.674	1.932		
	Conscientiousness	3.7 (0.7)	3.7 (0.7)	0.915	0.572	1.461	3.6 (0.7)		0.852	0.504	1.441		
	Extraversion	3.0 (0.8)	3.1 (0.7)	1.281	0.825	1.988	3.1 (0.6)		1.072	0.653	1.757		
	Agreeableness	3.6 (0.7)	3.7 (0.6)	1.227	0.755	1.993	3.8 (0.6)		1.387	0.803	2.396		
	Neuroticism	2.5 (0.8)	2.4 (0.8)	1.055	0.678	1.641	2.5 (0.9)		0.825	0.505	1.346		

^a^Italic text indicates a statistically significant variable.

## Discussion

### Principal Findings

This study sought to examine the psychosocial impact of COVID-19 on a sample of Australian adults. Natural language processing was applied to COVID-19 impact stories from more than 3000 Australian adults during the height of a national shutdown that was a core tenet of Australia’s COVID-19 containment strategy. The majority of participants’ impact stories (76.9%) were classified as having either neutral or negative sentiment. More than 70% of the main impacts detected through word frequency analysis fell into the life domains of behavioral regulation, environmental context and resources, and social influences. Statistically significant but small-magnitude associations of negative sentiment scores relative to positive scores with greater financial stress and lower satisfaction with life were identified for female, but not male, participants.

Knowledge of community sentiment during public health events can inform better health outcomes by providing insights into the psychosocial and environmental drivers of human behavior and in detecting unanticipated events or trends within the community [35]. Similar to previous research [36-38], our findings suggest an overall trend toward neutral and negative sentiment toward COVID-19. Neutral sentiment tends to indicate lower levels of concern about COVID-19 or minimal experiences of personal impact. It can also be the case that negative impacts are balanced out by positive ones to arrive at a net neutral result, which is common during the course of disruptive events [36]. This principle of sentiment balance was reflected in the data, whereby many participants reported that some aspects of their lives, such as homeschooling children, had become more stressful but that other life pressures, such as work commutes and social obligations or overall life busyness, had eased.

Approximately one-third of the impact stories were classified as reflecting negative sentiment. Among female participants, negative sentiment was associated with financial stress and subjective well-being. This finding is consistent with previous research, particularly evidence that COVID-19 is an independent source of psychological distress among Australian adults [12] and demonstration of the association between economic indicators (eg, financial distress, unemployment) and negative psychological outcomes, including increased prevalence of psychological disorders and suicide rates [39]. These findings bolster previous calls to increase mental health support, including evidence-based prevention programs, during national disasters [39]. Our findings suggest that initiatives are particularly needed that target individuals who are experiencing financial stress (eg, who have lost their job or are seeking income support) as a targetable correlate of negative sentiment and, potentially, mental health. However, it should be noted that the magnitude of these associations was small, which suggests that another confounder that was not measured may be influencing sentiment levels.

A further 23% of participants expressed a positive sentiment toward COVID-19. Local factors such as relatively low numbers of cases in Australia as well as the composition of the current research sample are potential explanations. From a global perspective, Australia’s COVID-19 response bore similarities with that of Thailand, South Korea, and Japan, including early and widespread intervention and very low rates of infection (<1000 cases per million) during 2020 [7]. This differs from countries in which responses were more anticipatory or reactive and that experienced high rates of infection (>4500 cases per million) as well as worse mortality outcomes during a similar time period [7]. It is likely that the proactiveness of Australia’s response, combined with a general tendency of Australians to trust health and science authorities [40], contributed to higher confidence and optimism for Australia’s recovery and therefore to the prevalence of neutral and positive sentiment. Epidemiological outcomes related to infection or mortality did not emerge as significant themes in the current data set, providing further indication that the main impacts were tied to the consequences of containment measures rather than the virus itself.

In terms of the nature of COVID-19 impacts, the life domain that was by far the most affected was behavioral regulation. Behavioral regulation is a critical psychological determinant of positive adaptation to change [39,41,42]; however, during the COVID-19 pandemic, these abilities are inhibited. Gym closures and the subsequent lost opportunities to exercise and socialize was a salient example in this data set, particularly for older respondents or those with injury or disability who require specialized equipment (eg, pools) or supervision. Similarly, our data suggest profound social and emotional implications associated with restrictions on movement within and between communities. For example, many individuals were restricted from providing their usual informal support and care to members of their social and familial networks. In some cases, the content of the COVID-19 impact stories reflects experiences of highly emotional and potentially traumatic individual experiences that reflect isolation, boredom, and frustration with the situation. Some pertinent examples included individuals who were unable to visit patients with dementia, individuals unable to support loved ones who were dying, and ongoing worry about the safety, security, and health of others.

Public health measures designed to counteract limitations on behavioral regulation during health crises are key to minimizing the psychosocial burdens of disaster response strategies. Across the board, COVID-19 has accelerated the digitization of services, with examples including the transition toward mixed-modality health care provision including telehealth appointments and other forms of remote communication and care and the growth of virtual gyms and coaching. These shifts have not only served as integral measures to cope with the COVID-19 pandemic but also increased access to services for all in the Australian community [43]. However, the digital divide should also be acknowledged, and face-to-face and assisted services should be maintained for those with limited access or capacity to use web-based services [44,45]. Evidence suggests that multisystem-level support programs are effective, including interventions targeted at individuals in the community, health care providers, and indirect interventions that address predictors of mental health programs [39].

Looking to the future, public sentiment toward the impact of COVID-19 will continue to play an important role in how we adapt to changing conditions and new public health measures, including, for example, the rollout of vaccines, localized self-isolation orders and outbreak containment measures, and digital contact tracing. The value of natural language processing is well known; however, the sources of data used in these applications have traditionally been limited to existing data sources, particularly social media (eg, Twitter tweets) and electronic health record data. Our findings highlight the feasibility and value of natural language processing with purpose-collected research data to answer unique research questions. Future studies are also needed that apply and compare different natural language processing techniques with research data. Topic modeling is one example that identifies latent topics based on the clustering of similar terms within a data set. These more advanced techniques, which take into account not only word frequency but also indications of proximity and other latent semantic patterns, may help to provide richer insights into the texts at hand.

### Strengths and Limitations

This is the first study to our knowledge to apply natural language processing to understand the psychosocial impacts of Australians during the COVID-19 pandemic using linked research data. Where possible, we used validated outcome measurement and analytical tools, including the robust Stanford CoreNLP analysis tool [26], to strengthen findings and allow comparisons with other studies. Limitations of the study included that the sample was biased toward women and relied upon self-report measures, which can be subject to social desirability bias. However, this can also be viewed as an advantage because the vast majority of natural language processing research draws upon social media data, such as tweets (from Twitter), as this is a common application of natural language processing. The demography of Twitter (and other social media platforms) is also biased and tends to overrepresent demographics such as urban residents, men, and people with specific interests in sports and politics [46,47]. Our sample, in comparison, is largely characterized by older women, who are not well represented in these platforms; this enabled us to capture a different angle on the same topic. Still, findings may have limited generalizability outside of this sample, and they therefore provide a snapshot of the experiences, and their potential implications, of one moderately sized sample of Australian adults. In future, it would be pertinent to repeat this process with different population segments to build a more complete picture of the psychosocial impacts of COVID-19.

### Conclusions

Our study identified a large prevalence of negative and neutral sentiment toward COVID-19 among a sample of Australian adults. The most common impacts of COVID-19 were in the life domains of behavioral regulation, environmental contexts and resources, social influences, and emotions. Our findings shed some light on the profound disruptions that the COVID-19 pandemic has created and continues to create in people’s routines and relationships, as well as some of the potential social and emotional consequences of these disruptions. Sentiment analysis as a deductive way to understand people’s experiences is of critical importance during significant public health events. Ongoing analysis of community sentiment is needed to inform optimum disaster response and preparedness measures.

## Appendix

Multimedia Appendix 1 Example quotations from COVID-19 impact stories.

## Abbreviations

VADER: Valence Aware Dictionary and Sentiment Reasoner
