# Supplementary material for: The Psychosocial Impacts of COVID-19 on a Sample of Australian Adults: Cross-sectional Survey and Sentiment Analysis
Source: JMIR Public Health Surveill. 2021 Jul 2;7(7):e29213. doi: 10.2196/29213 (PMC8274677; doi:10.2196/29213)
Supplement: Multimedia Appendix 1 [file publichealth_v7i7e29213_app1.docx]

| **Participant characteristics and sentiment score.** | *Quote from data* |
| --- | --- |
| **Female, 64 years old, 5/5 (very positive) sentiment score.** | *Actually, apart from not being able to hug and kiss my children and most importantly my grandchildren which was not ideal the COVID-19 outbreak seems to me to have bought people back to basics and to the now. That to me has been brilliant.* |
| **Female, 34 years old, 4/5 (positive) sentiment score***.* | *The COVID-19 outbreak has meant I am now working from home, which has been incredible for my mental health and wellbeing. I normally commute 2 hours per day, 3 times a week, and this was really taxing on me physically and emotionally. Working from home is so much more peaceful and stress-free. I am also the kind of person who benefits from working solo, so not being in the office environment has also been really positive for me. I was unable to get this opportunity before COVID-19, and only had 2 days a week approved to work closer to home. I now feel more confident to ask my employer for more days working from home.* |
| **Male, 52 years old, 3/5 (neutral) sentiment score**. | *I have been very fortunate to have little impact other than being locked down, working from home and adhering rigorously to social distancing. There has been stress with home schooling and all the family being cooped up, but benefits too, with all the family together and a focus on things we haven't done together for ages like jig saws and games.* |
| **Male, 27 years old, 2/5 (negative) sentiment score.** | *Lost my casual job in Australia. My overseas full-time job plans were cancelled. Now on jobseeker. Very depressing.* |
| **Female, 63 years old, 1/5 (very negative) sentiment score.** | *Exercise classes stopped. Gained a lot of Weight and not exercising. Now doing everything for older parents. Daughter going through a nasty separation and has moved back in with me with 2yr old & 4 yr old. Can’t get away from the stress or environment. Retired last June and have lost years of retirement funding*. |

*Supplementary Table 1.* Examples of very positive, positive, neutral, negative, and very negative COVID-19 impact stories.
